# Psychometric assessment of the Chinese adaptation of the patient participation scale targeting inpatients: a validation research

**DOI:** 10.3389/fpsyg.2024.1346131

**Published:** 2024-06-12

**Authors:** Rui Zhao, Mingshu Huo, Lei Wang, Sihan HuYan, Hongyu Li, Yan Cai

**Affiliations:** ^1^Department of Nursing, Jinzhou Medical University, Jinzhou, China; ^2^Department of Health and Nursing, Panjin Vocational and Technical College, Panjin, China; ^3^Institute of Medical Education, Jinzhou Medical University, Jinzhou, China

**Keywords:** patient participation, factor analysis, psychometric assessment, China, reliability analysis

## Abstract

**Objective:**

The objective of this research was to introduce, translate, and verify the Patient Participation Scale (PPS) within a Chinese context.

**Methods:**

We applied a combination of internal consistency testing, item analysis, exploratory factor analysis, and confirmatory factor analysis. The research involved 453 individuals, comprising both outpatients and inpatients, across three Jinzhou Medical University-affiliated hospitals in China. Additionally, a subgroup of 50 patients underwent a retest after a 2-week interval to assess reliability.

**Results:**

The adapted Chinese edition of the PPS included 21 items. Exploratory factor analysis identified four distinct factors, accounting for 66.199% of the total variance. Confirmatory factor analysis supported a suitable four-factor structure ( χ/df : 2.045, RMSEA: 0.048, GFI: 0.935, AGFI: 0.914, TLI: 0.958, CFI: 0.965, and PGFI: 0.712). The factor loadings corresponded to each item exceeded 0.6, the average variance extracted (AVE) exceeded 0.5, and the composite reliability (CR) exceeded 0.7. The correlation coefficients stayed below the square root of the AVE, demonstrated relatively favourable convergent and discriminant validity.The Chinese PPS edition demonstrated high internal consistency (Cronbach’s alpha: 0.919), with dimensional Cronbach’s alpha ranged from 0.732 to 0.918. Split-half as well as retest reliabilities were recorded at 0.737 and 0.864, respectively. The content validity index for the Chinese PPS edition stood at 0.974.

**Conclusion:**

The Chinese edition of the PPS emerges as a valid and reliable tool for assessing patient engagement in their own treatment as well as care, applicable in both inpatient as well as outpatient settings.

## Introduction

The advent of big data and advancements in internet communication technologies have significantly enhanced public access to healthcare information, fostering an environment where patients are increasingly involved in their own healthcare management (Epstein et al., 2010). This paradigm shift has been particularly crucial for chronic illness management, where patient empowerment in healthcare decision-making is a primary objective within the medical community (Singer et al., 2011). Since the 2005 London Declaration by the World Health Organization, patient participation has garnered growing scholarly interest (McCay et al., 2009; Barello et al., 2012; Coulter, 2012; Triberti and Barello, 2016).

The notion of patient participation, however, remains ambiguously defined both in China and internationally (Nilsson et al., 2019). Scholars from various regions conceptualize patient participation as the active involvement of patients in managing their health, encompassing activities like gathering health information and developing care plans, thus facilitating self-care processes (Gruman et al., 2010). Hibbard et al. (2004) interpreted patient activeness as the possession of knowledge, skills, and confidence for self-managing health and healthcare. Current evidence suggests that patient participation is integral to the process of delivering health services (Ding et al., 2019) and is essential for fostering patient motivation for self-management (Graffigna et al., 2019). Enhanced patient participation has been linked to improved health outcomes (Bombard et al., 2018), better quality of life (Selvin et al., 2021), and reduced healthcare costs (Weingart et al., 2011). The evolution of patient engagement has shifted the dynamics from a paternalistic model to a partnership-based approach in healthcare (Murali and Deao, 2019), suggesting a transition from patients as passive recipients to active contributors in the development and implementation of their care plans. Nonetheless, achieving optimal patient participation is challenging because of the significant knowledge disparity between healthcare professionals and patients, which often hinders effective communication (Angel and Frederiksen, 2015). The Patient Health Engagement (PHE) model, introduced by Graffigna et al. (2014), delineates the psychological stages of patient engagement, offering insights into enhancing patient involvement. Subsequent studies have shown that applying the PHE model can significantly improve active patient participation and adherence to medication regimens (Graffigna et al., 2017).

Song and Kim (2023), a researcher from Korea, developed an innovative scale for measuring patient engagement, comprising 4 dimensions and 21 items. The research team undertook a thorough literature review as well as qualitative interviews to comprehensively capture the concepts as well as nuances of patient engagement. The objective is to furnish healthcare professionals with a systematic instrument to evaluate the consistency of patient involvement in their health during both treatment and care. The scale effectively gauges patient engagement throughout the healthcare continuum, from outpatient treatment to inpatient care and eventual discharge. This approach ensures persistent patient involvement, not confined to specific intervals or sessions, fostering a robust partnership between patients and healthcare providers, which enhances treatment efficacy and patient satisfaction. Despite its significance, a similar patient participation scale tailored for the Chinese context is currently lacking. This research holds particular importance as Korea and China share cultural similarities as Asian nations. Our initial step involves translating the scale for application to both outpatients and inpatients in China, addressing the existing gap within patient engagement assessment, and serving as a basis for the development of a localized patient measurement tool. Furthermore, during the translation process, we aim to refine the scale’s content and structure by employing validation factor analysis, thereby reinforcing its validity as well as reliability.

## Methods

### Research design and participants

This research employed a cross-sectional survey approach, carried out from February to October 2023. The survey involved interactions with healthcare providers to evaluate patient involvement in their healthcare. According to Huo et al. (2023), the factor assessment required at least 10 respondents per item. Through convenience sampling, 453 participants, both outpatients and inpatients, were recruited from three hospitals, ensuring robust exploratory and confirmatory factor analysis.

Inclusion criteria for the subjects: (1) age ≥ 18 years; (2) currently receiving treatment in an outpatient clinic or hospital; (3) previous experience in outpatient or inpatient treatment; (4) voluntary consent to participate; and (5) ability to complete the questionnaire independently. Exclusion criteria for subjects: those with a history of neuropsychiatric disorders that severely affect cognitive functioning.

### Measurements

#### General demographics questionnaire

The research team developed a general demographic questionnaire for patients meeting the inclusion criteria. This questionnaire encompassed nine items: gender, age, relationship status, degree of education, place of living, occupation, diagnosis, and duration of the current medical visit.

#### Patient participation scale

Developed by Song and Kim (2023), this scale measures the degree of patient involvement in treatment and care in both outpatient and inpatient settings. Comprising 21 items across four dimensions, it utilizes a 5-point Likert scale (strongly agree: 5, somewhat agree: 4, neutral: 3, disagree: 2, strongly disagree: 1). The overall value of the scale runs from 21 to 105, with scores that are higher indicating greater patient involvement in their healthcare. The scale’s total Cronbach’s alpha coefficient is 0.92, denoting high reliability.

### Procedure

#### Data collection procedure

Researchers, trained uniformly, were organized into four groups of three and conducted recruitment at three Jinzhou Medical University-affiliated hospitals in Jinzhou City, Liaoning Province, China. Initially, 50 patients were selected for a preliminary survey using the post-translation scale, followed by a retest after 2 weeks to determine the scale’s retest reliability. In the main survey, 480 patients were initially chosen, of which 27 responses were deemed invalid, resulting in 453 valid questionnaires for assessment.

#### Translation and cross-cultural adaptation procedures

Upon receiving authorization from Professor Kim via email, the scale was translated following Brislin’s model (Khalaila, 2013). This involved an initial translation to Chinese by two Chinese English professors to create edition A. The researchers and professors then collaboratively refined this to produce edition B. Subsequently, two native English-speaking professors, unfamiliar with the original scale, translated edition B back into English. After further discussions, edition C was finalized. The researchers and translators then compared edition C with the original scale to complete the first draft of the Chinese Patient Participation Scale. Nine specialists in health promotion and psychometrics were consulted for content revision and cross-cultural adaptation (Navarro-Flores et al., 2018). Experts were asked to combine theoretical knowledge and clinical experience to propose modifications in terms of the clarity of the scale entries, the ease of understanding of the scale entries, the cultural background of the entries, their applicability, and the relevance of the entries, in order to ensure the questionnaire’s cultural applicability and content equivalence. At the same time, the relevance of each entry of Version C to the content of the study was evaluated and scored using a Likert 4-point scale, which was used to measure the content validity of the scale. The researcher collected and organised the results of the consultation, and based on the experts’ opinions, the group revised the content of the scale after discussion, and finally formed version D.

#### Data analysis procedure

SPSS 25.0 and AMOS 24.0 were used to evaluate the results. All statistical tests were two-sided, and a *p* value of less than 0.05 indicated statistical significance.

#### Item analysis

The item analysis employed the critical ratio method, ranking the overall scores of 453 patients and dividing them into the top 27% (high subgroup) and bottom 27% (low subgroup) categories. The differences between these groups were assessed using independent sample t-tests. Pearson correlation analysis examined the relationship between individual item scores and the total scale score. The criteria set (Zhang et al., 2022) included: (1) a critical ratio (CR) >3.0 for each item; and (2) a correlation coefficient > 0.4 between item scores and the total scale score.

#### Reliability analysis

The internal consistency of the scale was evaluated using the Cronbach’s α coefficient and the Spearman-Brown split-half reliability coefficient. To assess the scale’s stability, the retest reliability coefficient was used. The established criteria were as follows: (1) Cronbach’s α for the scale and its dimensions should be ≥0.7; (2) the split-half reliability coefficient should be ≥0.6; and (3) the retest reliability coefficient should be ≥0.7.

#### Validity analysis

To assess content validity, nine health promotion specialists were enlisted to evaluate every item on the scale, rating them as irrelevant, somewhat relevant, relevant, or highly relevant. Scores of 0 were assigned to irrelevant and somewhat relevant ratings, while relevant and highly relevant ratings received a score of 1. The Item-Content Validity Index (I-CVI)was calculated as the proportion of specialists who rated an item as 1, compared to the total number of specialists. The scale’s overall content validity score was the average of all I-CVI. An I-CVI ≥ 0.7 and an S-CVI ≥ 0.9 were required for validity. The scale’s factor structure was analyzed through exploratory factor analysis and confirmatory factor analysis. The Kaiser-Meyer-Olkin measure and Bartlett’s test of sphericity were employed in EFA, with a requisite KMO value >0.60 and a significant Bartlett’s test (p less than 0.05) (Pedreira et al., 2016). Principal component analysis extracted common factors, and highest variance rotation identified factors with initial eigenvalues >1. Scree plots, combined with the rotated factor structure, were used to determine the retention of common factors. CFA, conducted using AMOS, evaluated the fit of the validation model. AVE and CR were used to assess convergent validity, requiring AVE > 0.5 and CR > 0.7 for high validity. By contrasting the square root of AVE with the association coefficients involving observable variables, discriminant validity was investigated.If the AVE’s square root is greater than the correlation coefficient, indicating stronger discriminant validity (Wu et al., 2022).

#### Ethical approval

Our research adhered to the ethical standards of the 1964 Declaration of Helsinki (Holt, 2014). Informed consent was obtained from all participants, who completed anonymous questionnaires. The research protocol received approval from the Jinzhou Medical University’s Ethics Committee (JZMULL2023080).

## Results

### Demographic characteristics

This research encompassed 453 patients, comprising 232 males (51.2%) as well as 221 females (48.8%). The predominant age group was 41–59 years, representing 58.9% of the participants. A majority, 70.9%, were married. Tertiary education or higher was attained by 62.9% of patients. Rural residence was reported by 68.9% (312 patients), and 50.1% identified as freelancers. The majority (62.5%) were admitted for surgical reasons. Over half (52.1%) had a single doctor’s visit duration ranging from 5 to 10 days, as detailed within Table 1.

**Table 1:** Frequency distribution of demographic characteristics (*n* = 453).

Factors	Group	*n*	%
Age(years)	≤40	132	29.1
	41–59	267	58.9
	≥60	54	11.9
Sex	Male	232	51.2
	Female	221	48.8
Marital status	Married	321	70.9
	Unmarried	20	4.4
	Divorced/Widowed	112	24.7
Education level	Primary school or below	10	2.2
	Middle school	34	7.5
	High school and junior college	124	27.4
	College or above	285	62.9
Place of residence	city	141	31.1
	countryside	312	68.9
occupation	Freelance	227	50.1
	Retired	186	41.1
	Unemployed	40	8.8
Diagnosis	Inpatient medical treatment	75	16.6
	Inpatient surgery	283	62.5
	Outpatient emergency care	95	21.0
Length of single visit to the doctor	<5 days	105	23.2
	5–10 days	236	52.1
	≥11 days	112	24.7

### Translation and cross-cultural adaptation

The research team discussed, collated, and modified the entries of the scale in response to the results of the cultural survey as well as the pre-survey. The details are as follows.

(1) Some expressions were inappropriately worded and revised. For example, the term “HCP” (healthcare provider) in all entries of the original scale was modified to “healthcare provider” (including doctors, nurses, pharmacists, dietitians, etc.), so that patients would have a clearer understanding of what groups of people were included in the provision of relevant healthcare services to them during their hospitalisation or when they were receiving treatment on an outpatient basis.

(2) Some expressions did not conform to the Chinese language convention, and will be revised. For example, in entry 2: “If new symptoms appear or existing symptoms were changed, I would to inform the HCP” did not conform to the expression of our country’s patients who seek new medical treatment for themselves, so it should be changed to “I will notify my HCP if I develop a potential health problem or if there is a change in an existing health problem.” In entry 11: “I check my vital signs (blood pressure, pulse, temperature and respiratory rate) or test results and compare them with previous results,” the words “check” and “test results” does not fit the description of the health behaviour of monitoring vital signs by Chinese patients, so it is changed to “I will self-monitor my vital signs (blood pressure, pulse, temperature and respiratory rate) and compare them with previous results.” In entry 13: “I check that my treatment was carried out in accordance with the prescribed timetable,” the word “check” refers more to behavioural actions emanating from patients with a higher level of education, taking into account that there are also patients with a lower level of education who participate autonomously in self-management. So it was changed to “I monitor whether my treatment is carried out according to the prescribed timetable.”

As a result of the cultural adaptation and pre-survey, no scale entries were deleted or added. The results of the pre-survey showed that the scale was moderate and it took 5–10 min to complete one questionnaire, and the final version of the Chinese version of the Patient Participation Scale consisted of 4 dimensions and 21 items.

### Item analysis

The CR for the Chinese edition of the scale varied from 8.013 to 15.744 (*p less than* 0.01). Correlation coefficients (r) involving individual items as well as the overall scale score between 0.538 and 0.790 (*p less than* 0.01). Removal of each item individually resulted in total Cronbach’s α coefficients for the Chinese edition ranging from 0.950 to 0.954, remaining below the original coefficient. Consequently, all 21 items were retained, as depicted within Table 2.

**Table 2:** Item analysis for Chinese version of the patient participation scale.

Item	Critical ratio	Correlation coefficient between item and total score	Cronbach’s alpha if item deleted
PPS-1	15.744	0.777	0.951
PPS-2	13.141	0.707	0.951
PPS-3	10.903	0.681	0.952
PPS-4	12.832	0.733	0.951
PPS-5	8.013	0.538	0.954
PPS-6	14.577	0.727	0.951
PPS-7	11.837	0.699	0.952
PPS-8	13.548	0.685	0.952
PPS-9	9.152	0.658	0.952
PPS-10	9.677	0.684	0.952
PPS-11	14.217	0.771	0.951
PPS-12	12.112	0.728	0.951
PPS-13	10.282	0.709	0.952
PPS-14	14.391	0.766	0.951
PPS-15	14.823	0.764	0.951
PPS-16	14.426	0.750	0.951
PPS-17	14.154	0.740	0.951
PPS-18	13.207	0.745	0.951
PPS-19	14.839	0.778	0.951
PPS-20	12.109	0.722	0.951
PPS-21	13.908	0.790	0.950

### Reliability analysis

The Chinese edition of the Patient Participation Scale (PPS) exhibited a Cronbach’s α of 0.919, indicating high internal consistency. The scale’s split-half reliability coefficient stood at 0.737. For retest reliability, a subset of 50 patients was re-evaluated after 2 weeks, yielding a coefficient of 0.864. Cronbach’s alpha, split-half reliability coefficients and retest reliability coefficients for each dimension are presented in Table 3.

**Table 3:** Reliability analysis of the Chinese version of the patient participation scale.

The scale and its dimension	Cronbach’s alpha	Split-half reliability	Test–retest reliability
PPS	0.919	0.737	0.864
Performing autonomous self management activities	0.918	0.961	0.754
Sharing of information and knowledge	0.890	0.892	0.749
Establishing a mutual trust relationship	0.875	0.871	0.720
Partaking in the decision making process	0.732	0.735	0.645

### Validity analysis

#### Content validity analysis

Nine specialists assessed the content validity of the PPS’s Chinese translation. The I-CVI ranged from 0.889 to 1.000, and the S-CVI was calculated to be 0.974, as detailed within Table 4.

**Table 4:** Content validity analysis of the Chinese version of the Patient Participation Scale.

Item	Experts(score)									I-CVI
	1	2	3	4	5	6	7	8	9	
PPS-1	1	1	1	1	1	1	1	1	1	1.000
PPS-2	1	1	1	1	1	1	1	1	1	1.000
PPS-3	1	1	1	1	1	1	1	1	1	1.000
PPS-4	1	1	1	1	0	1	1	1	1	0.889
PPS-5	1	1	1	1	1	1	1	1	1	1.000
PPS-6	1	1	1	1	1	1	1	1	1	1.000
PPS-7	1	1	1	1	1	1	1	1	1	1.000
PPS-8	1	1	1	1	1	1	1	1	1	1.000
PPS-9	1	0	1	1	1	1	1	1	1	0.889
PPS-10	1	1	1	1	1	1	1	0	1	0.889
PPS-11	1	1	1	1	1	1	1	1	1	1.000
PPS-12	1	1	1	1	1	1	1	1	1	1.000
PPS-13	1	1	1	1	1	1	1	1	1	1.000
PPS-14	1	1	1	1	1	1	1	1	1	1.000
PPS-15	1	1	1	1	1	1	1	1	1	1.000
PPS-16	1	1	1	1	1	1	1	1	1	1.000
PPS-17	1	1	1	0	1	1	1	1	1	0.889
PPS-18	1	1	1	1	1	1	1	0	1	0.889
PPS-19	1	1	1	1	1	1	1	1	1	1.000
PPS-20	1	1	1	1	1	0	1	1	1	1.000
PPS-21	1	1	1	1	1	1	1	1	1	1.000

#### Exploratory factor analysis

**Figure 1:**
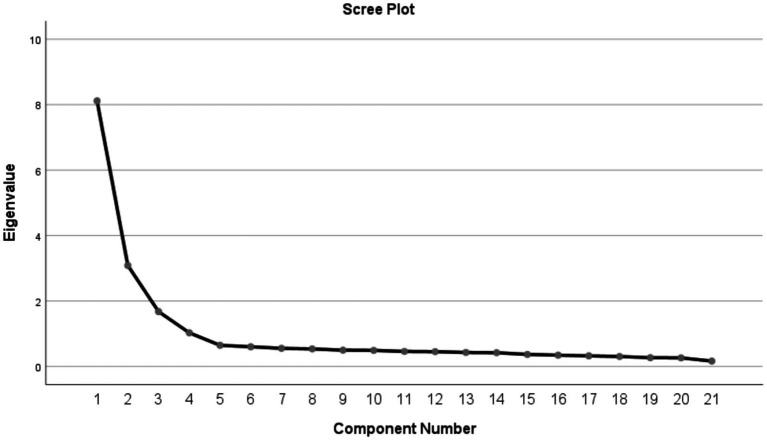
Scree plot of exploratory factor analysis for the Chinese version of the Patient Participation Scale.

The first half of the 226 questionnaires were used for exploratory factor analysis. The Kaiser-Meyer-Olkin measure for the PPS’s Chinese edition was 0.921, and Bartlett’s test of sphericity yielded a chi-square value of 5331.026 (*P* less than 0.001), validating its suitability for factor assessment. PCA extracted four common factors with initial eigenvalues exceeding 1. The scree plot (Figure 1) corroborated this finding, with no evidence of multifactor loading, as outlined within Table 5. Cumulatively, these factors accounted for 66.199% of the overall variance. Based on the content characteristics of the common factors, they are named separately as Performing autonomous self management activities, Sharing of information and knowledge, Establishing a mutual trust relationship, Partaking in the decision making process.

**Table 5:** Factor loading from exploratory factor analysis of the Chinese version of the patient participation scale.

Item	Factor 1	Factor 2	Factor 3	Factor 4
PPS-1	–	0.755	–	–
PPS-2	–	0.732	–	–
PPS-3	–	0.712	–	–
PPS-4	–	0.720	–	–
PPS-5	–	0.734	–	–
PPS-6	–	0.746	–	–
PPS-7	–	0.741	–	–
PPS-8	–	0.702	–	
PPS-9	–	–		0.798
PPS-10	–	–	–	0.766
PPS-11	0.823	–	–	–
PPS-12	0.809	–	–	–
PPS-13	0.789	–	–	–
PPS-14	0.717	–	–	–
PPS-15	0.796	–	–	–
PPS-16	0.774	–	–	–
PPS-17	0.756	–	–	–
PPS-18	–	–	0.778	–
PPS-19	–	–	0.779	–
PPS-20	–	–	0.782	–
PPS-21	–	–	0.795	–

#### Confirmatory factor analysis

**Figure 2:**
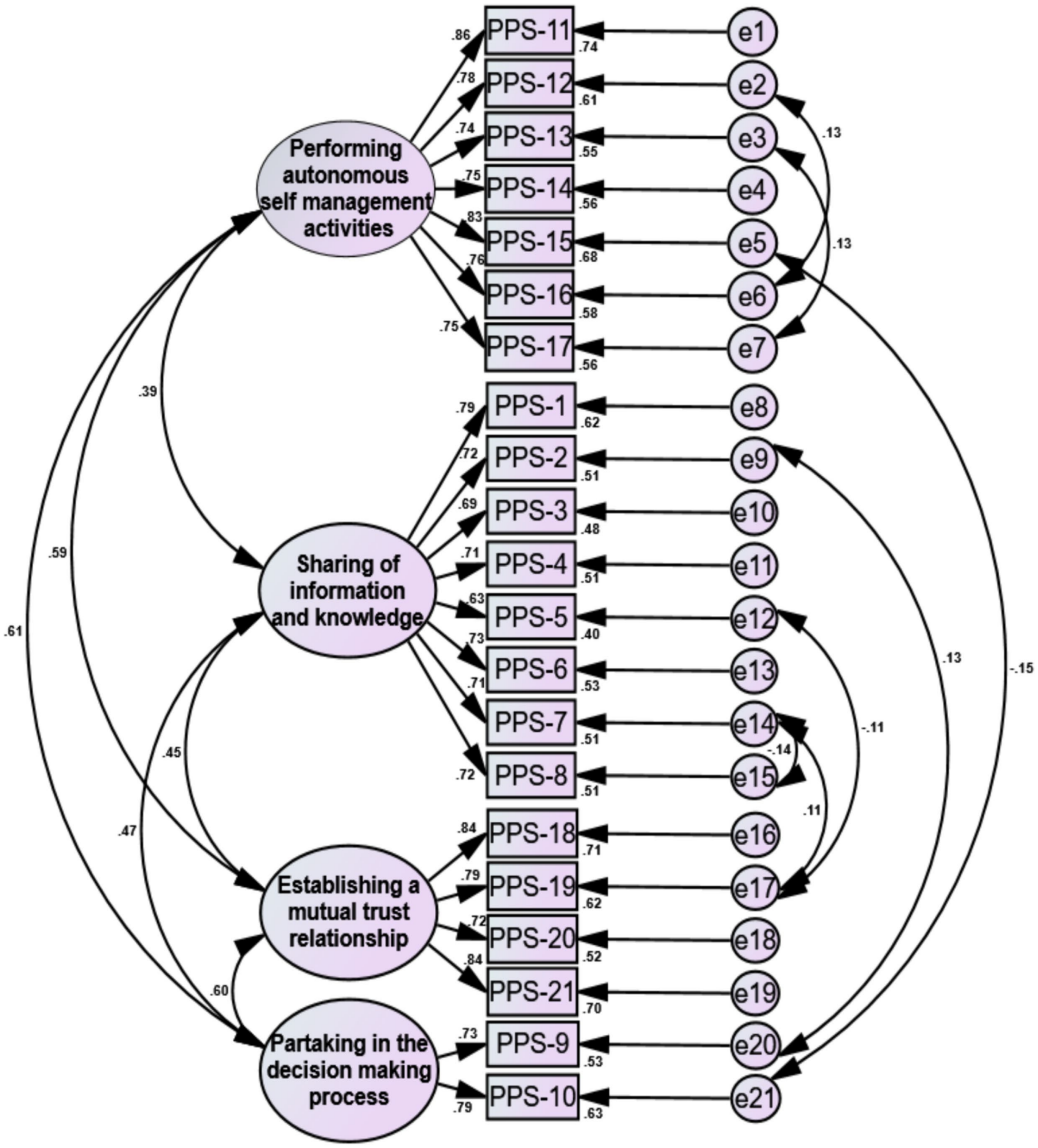
Standardized four-factor structural model of the Chinese version of the Patient Participation Scale.

The remaining one-half of the 227 questionnaires were used for the confirmatory factor analysis. A four-factor structural model was constructed using AMOS for confirmatory factor analysis with highest likelihood estimation. Based on the modification index, seven adjustments were made, e14-e7, e14-e15, e12-e17, e9-e20, e5-e21, e3-e7, and e2-e6.resulting in a final model fit ( χ/df : 2.045, RMSEA: 0.048, GFI: 0.935, AGFI: 0.914, TLI: 0.958, CFI: 0.965, PGFI: 0.712), as shown in Figure 2.

#### Convergent validity

Table 6 indicates that each factor loading exceeded 0.6, the AVE was above 0.5, and CR surpassed 0.7, confirming strong convergent validity.

**Table 6:** Convergent validity.

			Estimate	AVE	CR
PPS-11	<−--	Factor1	0.861	0.6135	0.9172
PPS-12	<−--	Factor1	0.784		
PPS-13	<−--	Factor1	0.744		
PPS-14	<−--	Factor1	0.749		
PPS-15	<−--	Factor1	0.827		
PPS-16	<−--	Factor1	0.760		
PPS-17	<−--	Factor1	0.750		
PPS-1	<−--	Factor2	0.787	0.5087	0.892
PPS-2	<−--	Factor2	0.715		
PPS-3	<−--	Factor2	0.689		
PPS-4	<−--	Factor2	0.713		
PPS-5	<−--	Factor2	0.634		
PPS-6	<−--	Factor2	0.730		
PPS-7	<−--	Factor2	0.711		
PPS-8	<−--	Factor2	0.718		
PPS-18	<−--	Factor3	0.842	0.6376	0.8752
PPS-19	<−--	Factor3	0.790		
PPS-20	<−--	Factor3	0.719		
PPS-21	<−--	Factor3	0.837		
PPS-9	<−--	Factor4	0.729	0.5801	0.7339
PPS-10	<−--	Factor4	0.793		

#### Discriminant validity

Significant correlations were observed among Factors 1, 2, 3, and 4 (*p* less than 0.001), as shown within Table 7. All coefficients of correlation were less than the AVE’s square root, indicating robust discriminant validity.

**Table 7:** Discriminant validity.

	F1	F2	F3	F4
F1	0.6135	–	–	–
F2	0.388^***^	0.5087	–	–
F3	0.586^***^	0.450^***^	0.6376	–
F4	0.609^***^	0.469^***^	0.605^***^	0.5801
Square root of AVE	0.7833	0.7132	0.7985	0.7616

The diagonal data are AVE values and the triangular data below them indicate the Pearson correlation coefficients between the dimensions, all *p* < 0.001.

## Discussion

This research involved the translation of the Patient Participation Scale (PPS) into Chinese and its cross-cultural adaptation, meticulously adhering to Brislin’s model and utilizing expert consultations (Khalaila, 2013). Nine specialists revised the initial translation to make it compatible with the linguistic nuances and context of our patient population. In the preliminary survey, 50 patients affirmed the Chinese PPS’s simplicity, clarity, and clinical relevance, commenting on its straightforward structure and comprehensible wording. The final Chinese PPS comprised 21 items across four dimensions. Item assessment revealed effective differentiation among the scale’s items (Huang et al., 2020), with each displaying moderate to high correlations with the overall scale. Removal of individual items did not significantly alter the Cronbach’s alpha value from that of the original scale, indicating the Chinese edition’s appropriateness and discriminative power.

Internal consistency and retest reliability are pivotal in evaluating a scale’s reliability (Anselmi et al., 2019). The Chinese PPS demonstrated a Cronbach’s alpha coefficient of 0.919, with dimension-specific coefficients ranging from 0.732 to 0.918, slightly surpassing the original edition’s consistency (Song and Kim, 2023). This suggests enhanced internal consistency in the Chinese adaptation. Furthermore, the split-half reliability coefficient, recorded at 0.737, corroborates this finding. Retest reliability, indicative of a test’s temporal stability and consistency (Leppink and Pérez-Fuster, 2017), was reflected in the Chinese PPS’s coefficient of 0.864. This confirms the stability as well as consistency of the Chinese edition as time passes by, underscoring its reliability.

This research evaluated the content validity of the PPS Chinese edition, engaging nine specialists for the evaluation. The results revealed I-CVI values ranging from 0.889 to 1.000 and a S-CVI of 0.974, surpassing the benchmark content validity values of 0.9 and 0.8 (Curtis and Keeler, 2021). This demonstrates the enhanced consistency and relevance of the scale’s content validity in the Chinese context. Furthermore, exploratory factor assessment (EFA) identified four distinct factors in the Chinese PPS, accounting for 66.199% of the cumulative variance. Factor loadings for each item exceeded 0.4, aligning with the factor attributions of the original scale (Song and Kim, 2023), indicating sound structural validity for the Chinese adaptation. Confirmatory factor assessment confirmed the acceptability of the four-factor model’s fit indices, further endorsing the structural validity of the Chinese PPS. The AVE for each dimension exceeded 0.5, and the CR was above 0.7, indicating a high correlation among items within each dimension, effectively measuring distinct aspects of patient participation. This demonstrates robust convergent validity (Sharif Nia et al., 2021). Additionally, The inter-dimension coefficients of correlation were all less than the AVE’s square root, suggesting the Chinese PPS’s efficacy in distinctly capturing the four attributes of patient engagement while excluding confounding factors (Wu et al., 2022), thereby confirming its strong discriminant validity.

In conclusion, the Chinese adaptation of the Patient Participation Scale (PPS) demonstrates both content and structural validity for clinical research and application. This edition effectively quantifies patient engagement in their own treatment and care processes, applicable in both inpatient and outpatient contexts. The Chinese version of the PPS contains 4 dimensions and a total of 21 entries, which more comprehensively cover patients whether they are treated during hospitalization or in outpatient clinics, and such participation is of great significance and value in clinical treatment. By actively participating in medical decision-making, patients are able to better understand the nature of the disease, treatment options, and possible risks and benefits; in addition to this, e.g., patients’ regular monitoring of health indicators, taking medication on time, and maintaining good lifestyle habits not only improve the execution of treatment but also enhance their sense of responsibility and self-efficacy for their health. As participants in treatment, patients are able to provide valuable information about their medical history, symptoms, and treatment response, which is crucial for doctors to make a correct diagnosis and formulate effective treatment plans; at the same time, doctors can also provide patients with relevant health knowledge and treatment information, which can help them better understand the disease and the treatment process, and improve the degree of cooperation and the effectiveness of treatment. Patient participation in the decision-making process of clinical treatment can make the treatment plan closer to their individualized needs and preferences and enhance the relevance and sustainability of the treatment; by discussing the treatment choices, risks, and expected outcomes with doctors, patients can better understand their right to choose and the impact of their decision-making and enhance their confidence in and adherence to the treatment plan. When patients are involved in the treatment decision-making and management process, they feel the doctor’s respect for their needs and rights, thus establishing a closer and more positive partnership; at the same time, doctors will also value their opinions and needs more because of the patients’ participation, further enhancing the trust and consensus between the two parties, which will be conducive to the conduct and success of long-term treatment. When patients are involved in the treatment decision-making and management process, they feel the doctor’s respect for their needs and rights, thus establishing a closer and more positive partnership; at the same time, doctors will also value their opinions and needs more because of the patients’ participation, further enhancing the trust and consensus between the two parties, which will be conducive to the conduct and success of long-term treatment.

## Conclusion

The introduction and cultural adaptation of the Patient Participation Scale in China have been accomplished, confirming its psychometric robustness in various healthcare settings. This scale serves as a valuable tool for healthcare professionals, particularly nurses, to devise educational programs and interventions aimed at fostering patient involvement. Enhancing patient participation in treatment and care during their hospital and clinic visits can significantly improve healthcare outcomes. However, there are some limitations in this study, due to the limited conditions of the study, insufficient representativeness and a single source of sample, the next step will be to further expand the sample size and take samples from more departments in more hospitals across the country, to further validate the applicability and reliability of the scale.

## Data availability statement

The original contributions presented in the study are included in the article/Supplementary material, further inquiries can be directed to the corresponding authors.

## Ethics statement

The studies involving humans were approved by Jinzhou Medical University (Ethical approval number: JZMULL2023080). The studies were conducted in accordance with the local legislation and institutional requirements. The participants provided their written informed consent to participate in this study. Written informed consent was obtained from the individual(s) for the publication of any potentially identifiable images or data included in this article.

## Author contributions

RZ: Validation, Methodology, Investigation, Data curation, Writing – original draft. MH: Writing – original draft, Conceptualization. LW: Writing – original draft, Investigation. SH: Writing – original draft, Data curation. HL: Writing – review & editing, Validation, Supervision. YC: Writing – review & editing, Validation, Supervision, Project administration.
